# Perioperative risk prediction in the era of enhanced recovery: a comparison of POSSUM, ACPGBI, and E-PASS scoring systems in major surgical procedures of the colorectal surgeon

**DOI:** 10.1007/s00384-018-3141-4

**Published:** 2018-08-04

**Authors:** Nigel M. Bagnall, Edward T. Pring, George Malietzis, Thanos Athanasiou, Omar D. Faiz, Robin H. Kennedy, John T. Jenkins

**Affiliations:** 1 Department of Surgery and Cancer, Imperial College, London, Paddington W2 1NY UK; 2 Department of Surgery, St Marks Hospital, Watford Road, Harrow, Middlesex HA1 3UJ UK; 3 The George Davies Research Fellowship, St Marks Hospital, Watford Road, Harrow, Middlesex HA1 3UJ UK

**Keywords:** Enhanced recovery, Colorectal surgery, Preoperative risk, ERAS, ERP

## Abstract

**Purpose:**

This study aims to determine whether traditional risk models can accurately predict morbidity and mortality in patients undergoing major surgery by colorectal surgeons within an enhanced recovery program.

**Methods:**

One thousand three hundred eighty patients undergoing surgery performed by colorectal surgeons in a single UK hospital (2008–2013) were included. Six risk models were evaluated: (1) Physiology and Operative Severity Score for the enumeration of Mortality and Morbidity (POSSUM), (2) Portsmouth POSSUM (P-POSSUM), (3) ColoRectal (CR-POSSUM), (4) Elderly POSSUM (E-POSSUM), (5) the Association of Great Britain and Ireland (ACPGBI) score, and (6) modified Estimation of Physiologic Ability and Surgical Stress Score (E-PASS). Model accuracy was assessed by observed to expected (O:E) ratios and area under Receiver Operating Characteristic curve (AUC).

**Results:**

Eleven patients (0.8%) died and 143 patients (10.4%) had a major complication within 30 days of surgery. All models overpredicted mortality and had poor discrimination: POSSUM 8.5% (O:E 0.09, AUC 0.56), P-POSSUM 2.2% (O:E 0.37, AUC 0.56), CR-POSSUM 7.1% (O:E 0.11, AUC 0.61), and E-PASS 3.0% (O:E 0.27, AUC 0.46). ACPGBI overestimated mortality in patients undergoing surgery for cancer 4.4% (O:E = 0.28, AUC = 0.41). Predicted morbidity was also overestimated by POSSUM 32.7% (O:E = 0.32, AUC = 0.51). E-POSSUM overestimated mortality (3.25%, O:E 0.57 AUC = 0.54) and morbidity (37.4%, O:E 0.30 AUC = 0.53) in patients aged ≥ 70 years and over.

**Conclusion:**

All models overestimated mortality and morbidity. New models are required to accurately predict the risk of adverse outcome in patients undergoing major abdominal surgery taking into account the reduced physiological and operative insult of laparoscopic surgery and enhanced recovery care.

**Electronic supplementary material:**

The online version of this article (10.1007/s00384-018-3141-4) contains supplementary material, which is available to authorized users.

## Introduction

Colorectal surgery carries inherent perioperative risks, particularly for elderly patients with multi-morbidity [1]. Accurate risk stratification is essential to inform discussions with patients in order to facilitate informed consent, to enable surgical planning, and to anticipate the need for high dependency and intensive care support. Several models have been devised to predict postoperative mortality following general surgical operations.

In 1991, Copeland and colleagues developed the Physiology and Operative Severity Score for the enumeration of Mortality and morbidity (POSSUM) to allow risk adjustment of operations to enable comparative audit of different centers [2]. However, this model was shown to overestimate mortality in patients undergoing low-risk procedures and led the development of a recalibrated version termed Portsmouth-POSSUM (P-POSSUM) using a dataset of 10,000 general surgical operations [3]. Both POSSUM and P-POSSUM incorporate data from the same 12 physiological and 6 operative parameters.

Specific models to determine risk in colorectal surgery have also been created. In 2004, a risk prediction model termed ColoRectal POSSUM (CR-POSSUM) was developed using prospective data collected from 6883 colorectal operations from 15 UK hospitals between 1993 and 2001 [4]. This model had the advantage of only requiring six physiological variables and four operative parameters to predict 30-day mortality. However, once again this model has been shown to have limitations when predicting outcome in older patients. More recently, a French research group recalibrated the original POSSUM algorithm using data from 1186 patients aged 65 or over undergoing colorectal surgery across 41 hospitals, to generate an elderly-specific risk score (E-POSSUM) [5]. To date, POSSUM and Elderly POSSUM are the only scores validated to predict perioperative mortality and morbidity.

The Association of Coloproctology of Great Britain and Ireland (ACPGBI) developed a risk prediction model for patients undergoing colorectal cancer surgery requiring data on five variables: age, cancer resection, ASA grade, Dukes’ stage, and operative urgency [6, 7]. Although developed from a UK-based population, POSSUM and ACPGBI risk scores have been externally validated and shown to have good predictive power in studies of patients undergoing colorectal surgery in the Netherlands [8] and China [9].

In Japan, a mortality prediction risk score was developed termed Estimation of Physiological Ability and Surgical Stress (E-PASS) [10] using parameters to assess the health status of the patient (ASA score, co-morbidities, performance status) and the stress of surgery (blood loss, operation time, open or laparoscopic surgery). E-PASS has been validated for patients undergoing elective general surgery [11, 12] and colorectal surgery [13]. E-PASS has not been validated in a UK-based population.

All these risk models were derived from analyzing large datasets of patients undergoing open surgery with standard perioperative care. Advances in surgical practice with the increased use of a laparoscopic approach and enhanced recovery pathways may impact on the relevance and value of this data set. Senagore and colleagues demonstrated that laparoscopic surgery reduces true morbidity and mortality compared to the POSSUM and P-POSSUM values. Furthermore, a reduction of the operative severity from major to minor with the resultant reduction of severity score by three corrected the overprediction of morbidity by POSSUM and mortality by P-POSSUM in laparoscopic colectomies [14]. Enhanced recovery in turn has been demonstrated to minimize the stress response to surgery thus reducing complications and expediting the recovery process as a result [15, 16]. We hypothesize that existing risk prediction models may overestimate the incidence of mortality and morbidity following elective colorectal surgery with enhanced recovery care. The aim of this study was to compare the predicted mortality and morbidity in each risk prediction model with the true mortality and morbidity of a large prospective cohort of patients who underwent colorectal surgery with enhanced recovery care.

## Methods

Data was obtained from a prospectively maintained database of consecutive patients undergoing colorectal surgery with enhanced recovery care between 2008 and 2014. Ethical approval and individual written patient consent was not required because data were anonymized to the researchers which conforms to NHS research guidelines. Data on age, gender, surgical approach (laparoscopic, open, or converted), American Society of Anesthesiology (ASA) grade, and procedure type were prospectively collected together with data necessary to calculate the risk prediction scores. Complications were entered prospectively and missing data added from case notes by either the operating surgeon or a trained specialist nurse. These were defined according to POSSUM [17] and by the Clavien-Dindo classification [18]. Mortality was defined as any death that occurred during the first 30 days and within the hospital admission if longer than 30 days. The predicted risk of mortality and morbidity was determined using POSSUM model. In addition, we calculated predicted mortality using P-POSSUM, CR-POSSUM, and E-PASS for all patients. Normal values were substituted for missing data as per the method adopted by Senagore et al. in 2004 [19]. A separate analysis was performed to determine the accuracy of each model to predict outcome in patients undergoing surgery for colorectal cancer, inflammatory bowel disease, and other benign colorectal conditions. We also analyzed the accuracy of the ACPGBI score to predict mortality for all patients undergoing surgery for colorectal cancer. Finally, we determined the risk of mortality and morbidity in a subgroup of patients aged 70 years or more using POSSUM and E-POSSUM models.

## Description of how to calculate risk scores

POSSUM, P-POSSUM, CR-POSSUM, and ACPGBI scores were calculated using the method described by Copland et al., Prytherch et al., Tekkis et al., and Ferjani et al., respectively [2–4, 7]. Elderly POSSUM scores were derived using the algorithm by Tran Ba Loc et al. 2009 [5], and E-PASS scores by the method described by Haga et al. [13]. The algorithms are provided in appendices 1 and 2.

## Statistical analysis

To determine the validity of the risk prediction models, discrimination and calibration of each model were calculated. Discrimination is the ability of the model to assign a higher probability of death to the patients who actually died than those who were alive 30 days after surgery. This is determined by generating receiver-operating characteristic curves (ROC), with sensitivity (*y*-axis) plotted against specificity (*x*-axis). The area under the curve (AUC) of < 0.7 indicates poor discrimination, 0.7–0.8 indicates fair discrimination, and 0.8–1 good to excellent discrimination. AUC was calculated with 95% confidence intervals and compared using non-parametric paired tests in the method described by DeLong et al. (1988) [20]. The Bonferroni correction was used to adjust for pairwise comparisons.

Calibration is the accuracy of the model to predict the risk of death or complication for an individual patient. The estimated probability of death was calculated for each model and ranked into five equal groups of increasing operative mortality. The true mortality (observed) is then compared with the predicted mortality (expected) in each group. The observed/expected ratio of 1 is perfect accuracy, a ratio < 1 indicates overprediction of mortality rate, and a ratio of > 1 indicates underestimation. A Hosmer-Lemeshow C goodness of fit test is then used to generate a Chi squared test comparing the observed and expected outcome of all patients. A well-calibrated model in which the observed and expected mortality are similar would have a low Hosmer-Lemeshow value and *P* value > 0.05.

## Statistical analysis

All statistical analyses were performed using SPSS version 18.

## Results

We analyzed data from 1380 consecutive patients who underwent elective colorectal surgery performed by three surgeons within an enhanced recovery program. 0.03% of overall data was missing from the database, and normal values were substituted. Seven hundred sixty-four patients were male (55.4%) and 616 patients were female (44.6%). The median age was 62 years (interquartile range 43–72 years). Five hundred sixty-eight patients (41.2%) underwent resection for colorectal cancer (CRC), 217 patients (15.7%) underwent resection for inflammatory bowel disease (IBD), and 595 patients (43.1%) underwent surgery for other benign colorectal conditions (BC). One thousand fifty-six patients (76.5%) had surgery performed by laparoscopic technique, 238 patients (17.2%) had open surgery, and 86 patients (6.23%) initially underwent laparoscopic surgery but required conversion to open. The characteristic of the patients in the study are summarized in appendix 5 supplementary data table (a).

The combined 30-day postoperative and in hospital mortality rate was 11/1380 (0.797%). Seven patients died following CRC surgery: five cardiac failure and two pneumonia/respiratory failure. Four patients undergoing BC procedures also died: two from cardiac events and two respiratory failure. No deaths occurred in the group undergoing surgery for IBD.

The overall 30-day major complication rate (Clavien score of ≥ 3) was 143/1380 (10.4%): 69/568 patients (13.8%) with CRC, 30/217 patients (14.6%) with IBD, and 44/595 patients (10.2%) with other BC. Using the original POSSUM definition, the overall complication rate was 164/1380 (11.9%). The breakdown is presented in appendix 6 supplementary data table (b).

## Performance of mortality prediction models in the entire dataset

**Fig. 1:**
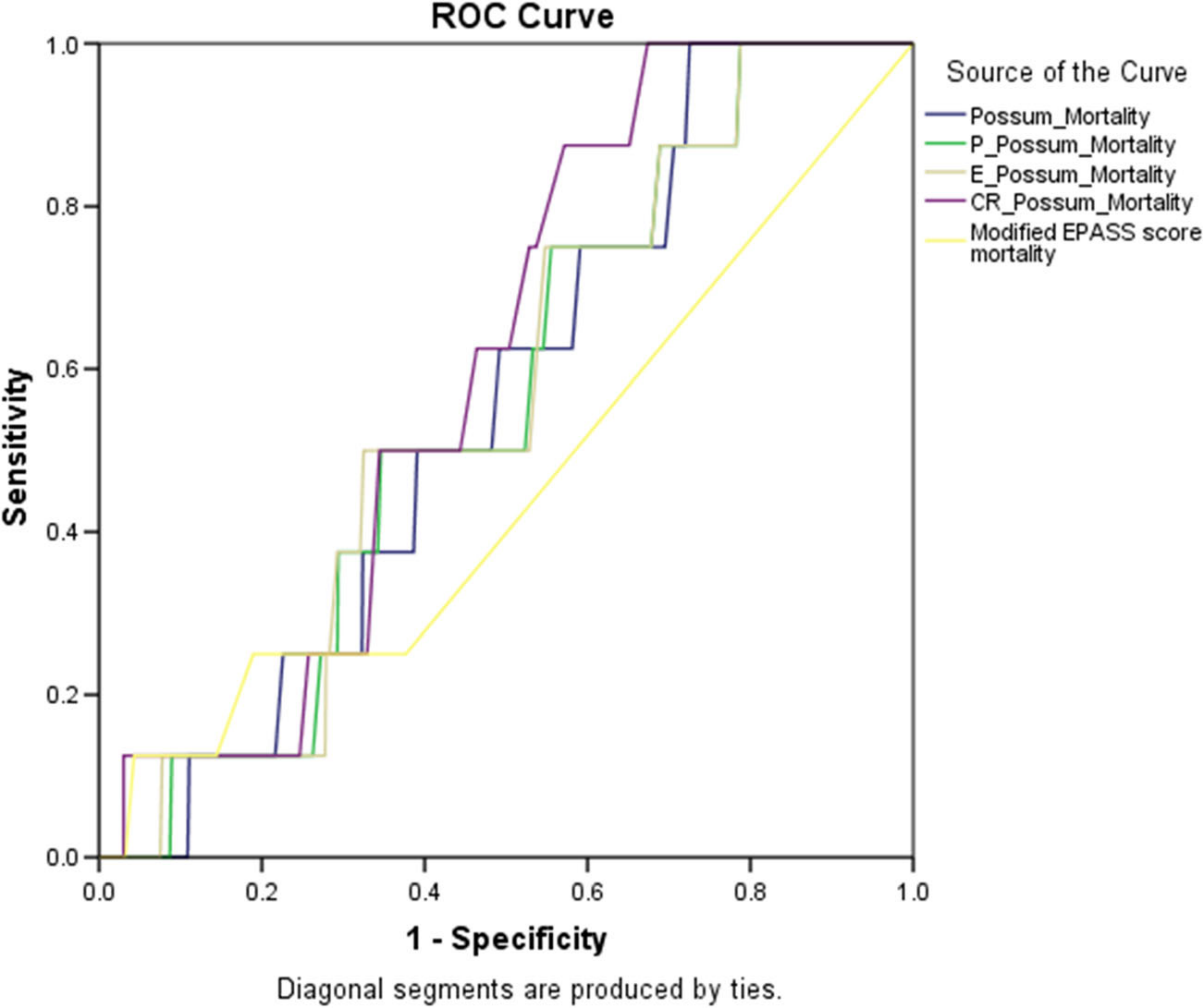
Receiver Operator Characteristic curves for mortality in entire cohort: comparing POSSUM, P-POSSUM, CR-POSSUM, E-POSSUM, ACPGBI, E-PASS

Table 1 and Fig. 1 show that all mortality risk prediction models demonstrated poor discrimination ability when applied to our dataset of all patients undergoing colorectal surgery. CR-POSSUM had the highest AUC value of 0.607 (95% CI 0.476–0.738) and best performing calibration of all models (Hosmer-Lemeshow 3.601, *P* = 0.891). All models significantly overpredicted perioperative mortality determined by observed by expected ratios of < 1.

**Table 1:** Discrimination and calibration of the studied scores for predicting mortality in colorectal patients

	O	E	O:E	Discrimination		Calibration		
				AUC (95%CI)	P*	H-L	df	P**
POSSUM	11	117	0.09	0.558 (0.412–0.703)	0.575	9.362	8	0.313
P-POSSUM	11	30	0.37	0.557 (0.404–0.711)	0.576	6.343	8	0.609
CR-POSSUM	11	98	0.11	0.607 (0.476–0.738)	0.296	3.601	8	0.891
E-POSSUM	11	40	0.28	0.561 (0.406–0.717)	0.551	11.863	8	0.157
E-PASS	11	41	0.27	0.458 (0.25–0.667)	0.683	1.884	3	0.597

*O* observed 30-day mortality rate, *E* expected 30-day mortality rate, *O*:*E* observed to expected mortality ratio (value closest to 1 indicate more accurate model)

Discrimination is measured by area under receiver-operator characteristic curve (AUC) with 95% confidence intervals, *P of the test of difference between scores for the AUC as described by DeLong et al.

Calibration is measured by the Hosmer-Lemeshow test: smaller H-L Chi square values, and larger **P values represent better model calibration

## Performance of mortality predictive models in subgroups

All models demonstrated poor discrimination to predict mortality in patients undergoing colorectal surgery for CRC (Table 2), benign colorectal disease (Table 3), or in patients aged ≥ 70 years or more (Table 4). Once again CR-POSSUM had the highest AUC values of 0.618, 0.565, and 0.592, respectively. Of the models tested, ACPGBI score most accurately predicted mortality for an individual patient having surgery for colorectal cancer (H-L 2.357, *P* = 0.938). Whereas, E-PASS most closely predicted mortality for colorectal patients with benign disease (H-L 2.432, *P* = 0.488) and individuals aged ≥ 70 years old (H-L 2.36, *P* = 0.501). As no deaths occurred in any patients undergoing surgery for IBD, we could not test the models on this subgroup.

**Table 2:** Discrimination and calibration of the studied scores for predicting mortality in patients undergoing surgery for colorectal cancer

	O	E	O:E	Discrimination		Calibration		
				AUC (95%CI)	P*	H-L	df	P**
POSSUM	7	50	0.14	0.574 (0.345–0.803)	0.610	8.395	8	0.396
P-POSSUM	7	13	0.54	0.566 (0.307–0.825)	0.649	11.868	8	0.157
CR-POSSUM	7	43	0.16	0.618 (0.370–0.867)	0.416	6.992	8	0.537
E-POSSUM	7	17	0.42	0.569 (0.305–0.834)	0.634	10.685	8	0.220
E-PASS	7	15	0.47	0.471 (0.153–0.788)	0.859	3.129	3	0.372
ACPGBI	7	25	0.28	0.406 (0.114–0.667)	0.516	2.357	7	0.938

Same legend as Table 1

**Table 3:** Discrimination and calibration of the studied scores for predicting mortality in patients undergoing surgery for other benign diseases

	O	E	O:E	Discrimination		Calibration		
				AUC (95%CI)	P*	H-L	df	P**
POSSUM	4	52	0.07	0.520 (0.336–0.705)	0.889	5.687	8	0.682
P-POSSUM	4	13	0.31	0.524 (0.355–0.692)	0.870	5.673	8	0.684
CR-POSSUM	4	41	0.10	0.565 (0.478–0.652)	0.656	10.799	8	0.213
E-POSSUM	4	17	0.24	0.529 (0.367–0.691)	0.843	11.921	8	0.155
E-PASS	4	10	0.4	0.438 (0.159–0.718)	0.672	2.432	3	0.488

Same legend as Table 1

**Table 4:** Discrimination and calibration of the studied scores for predicting mortality in colorectal patients aged 70 years and older

	O	E	O:E	Discrimination		Calibration		
				AUC (95%CI)	P*	H-L	df	P**
POSSUM	8	40	0.2	0.544 (0.340–0.748)	0.713	10.095	8	0.250
P-POSSUM	8	11	0.73	0.538 (0.329–0.747)	0.752	4.423	8	0.817
CR-POSSUM	8	35	0.23	0.592 (0.425–0.758)	0.443	8.623	8	0.375
E-POSSUM	8	14	0.57	0.539 (0.333–0.746)	0.741	9.698	8	0.287
E-PASS	8	12	0.67	0.374 (0.177–0.570)	0.289	2.36	3	0.501

Same legend as Table 1

## Morbidity prediction

**Fig. 2:**
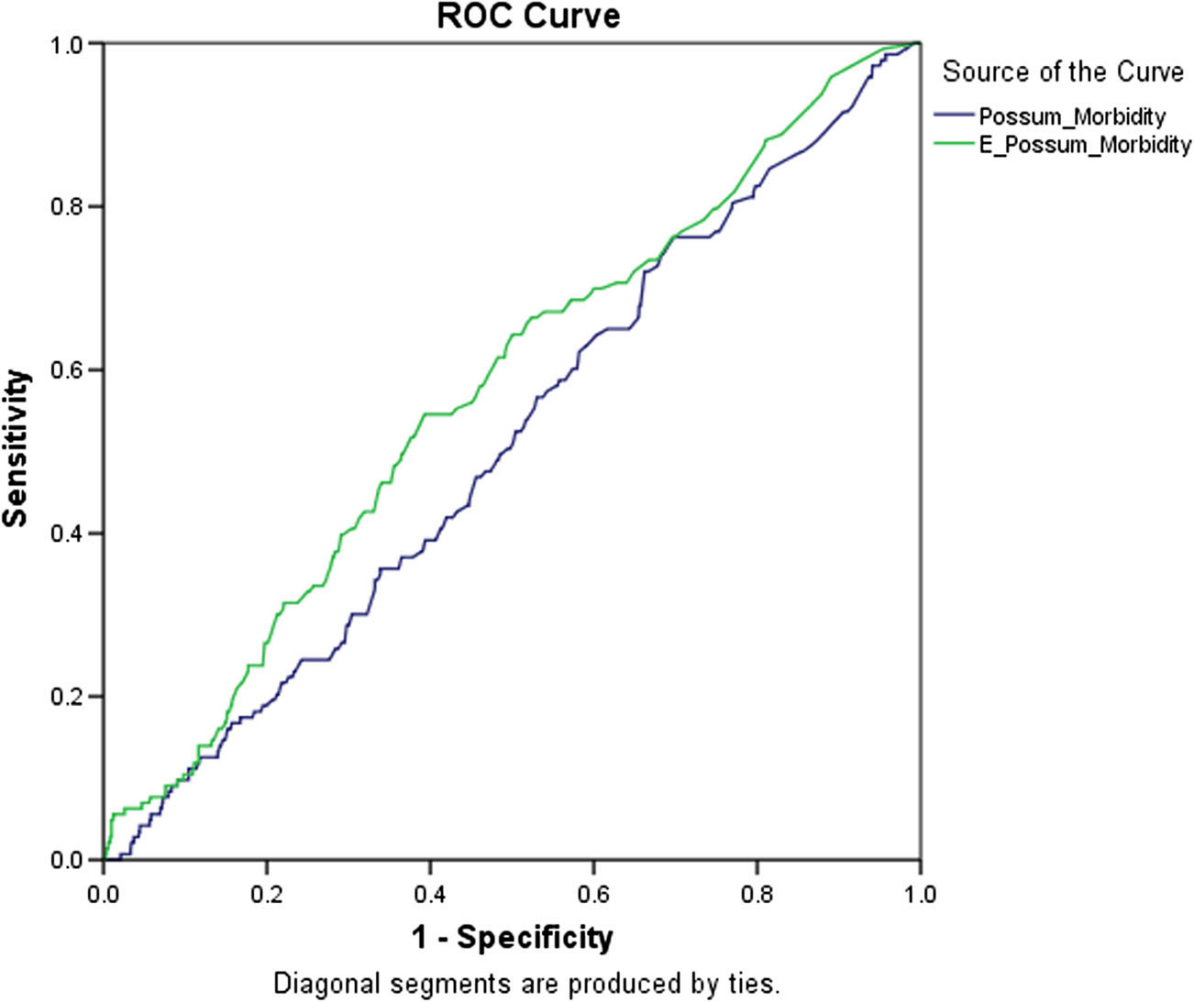
Receiver Operator Characteristic curves for mortality: comparing POSSUM and E-POSSUM for morbidity

In enhanced recovery patients, both POSSUM and E-POSSUM models demonstrated poor discrimination to predict major complications in the first 30 days following surgery (Table 5, Fig. 2). This was also seen when analyzing subgroups by type of surgery and age. E-POSSUM had the most reliable discrimination (AUC 0.572, *P* = 0.005) and calibration (H-L = 7.962, *P* = 0.437) of all models to predict complications in individual patients aged 70 years or more (Table 6).

**Table 5:** Discrimination and calibration of the studied scores for predicting major complications (Clavien ≥ 3) in colorectal patients

	O	E	O:E	Discrimination		Calibration		
				AUC (95%CI)	P*	H-L	Df	P**
POSSUM (all)	143	451	0.32	0.510 (0.462–0.558)	0.693	14.869	8	0.062
POSSUM (cancer)	69	189	0.37	0.529 (0.458–0.600)	0.432	7.067	8	0.529
POSSUM (IBD)	30	84	0.36	0.451 (0.340–0.562)	0.392	6.218	8	0.514
POSSUM (benign)	44	195	0.26	0.519 (0.435–0.602)	0.678	3.689	8	0.884
E-POSSUM (all)	143	439	0.33	0.572 (0.524–0.620)	0.005	7.962	8	0.437
E-POSSUM in ≥ 70 years old	48	161	0.30	0.531 (0.443–0.619)	0.488	9.956	8	0.268

Same legend as Table 1

**Table 6:** Discrimination and calibration of the studied scores for predicting complications (original POSSUM criteria) in colorectal patients

	O	E	O:E	Discrimination		Calibration		
				AUC (95%CI)	P*	H-L	Df	P**
POSSUM (all)	164	451	0.36	0.525 (0.479–0.571)	0.296	11.459	8	0.177
E-POSSUM (all)	164	439	0.37	0.645 (0.601–0.689)	< 0.001	7.359	8	0.498
E-POSSUM in ≥ 70 years old	61	161	0.38	0.600 (0.522–0.678)	0.013	5.443	8	0.709

431 patients older than 70

Same legend as Table 1

## Discussion

This study is the first to evaluate the validity of perioperative risk prediction models in patients who have undergone surgery within a rigorously followed enhanced recovery care program. All the models tested in our study overpredicted mortality and morbidity. Previous studies of patients undergoing colorectal surgery within an enhanced recovery program have been shown to have fewer complications and faster recovery than those managed with conventional care [21].

These risk stratification models are now used routinely in clinical care, especially P-POSSUM and CR-POSSUM. Both patients and clinicians are making clinical decisions guided by the results extrapolated from online calculators, which use these tools. This study’s findings therefore have significant implications when counseling patients regarding perioperative risk in order to guide treatment decisions and help to determine the level of postoperative care required. Surgeons who have adopted laparoscopic techniques and enhanced recovery principles should be aware of the limitations of the current risk models available. Importantly, emphasis should be made on cautious interpretation of the results of these calculations and the dangers associated with using them for risk prediction of mortality in individual patients. Additionally, there has been a recent focus on risk stratification in order to evaluate hospital and surgeon specific complications rates as part of quality improvement and safety initiatives. We have demonstrated the relatively poor reliability of current risk prediction models in patients undergoing colorectal surgery with enhanced recovery care. Indeed one can argue that both POSSUM and P-POSSUM, which were initially designed for comparative audit [2, 3], are being used inappropriately in clinical practice and that the historical data from which they are derived is outmoded and even redundant in relation to the recent advances in surgical technique and perioperative care.

The traditional risk models evaluated in this study are based on the multivariate regression analysis of perioperative variables recorded in large numbers of patients who have undergone open surgical procedures and not managed with enhanced recovery care protocols. Due to the low mortality associated with ERAS, we estimate a minimum dataset of approximately 10,000 patients with 100 deaths (10 deaths per model independent variables) would be required in order to maintain model integrity [22, 23]. This would then require further validation with data from other international institutions practicing ERAS.

The models were also poor at predicting mortality when analyzing patients stratified by age or disease pathology. Therefore new models need to include patients of all ages and encompassing a wide breadth of indications for surgery, if they can be applicable to these subgroups. Furthermore, new outcome predictive variables are becoming readily available, for example, sarcopenia, which can be demonstrated on preoperative computer tomography, has been shown to have a negative effect on postoperative outcomes [24–27].

There are several limitations in our study. Firstly, this is a single institution study based in a tertiary colorectal specialist center, and the results may not be generalizable to other hospitals who have adopted ERAS care protocols. Second, the performance of any mortality predictive model is dependent on the number of events, and in particular, deaths. The mortality and morbidity rates of patients undergoing surgery with ERAS were relatively small and therefore validation through a much larger dataset is required as described above. Third, we substituted normal values for missing data, although this represented less than 0.03% of the overall data. Finally, in order to confirm our hypothesis that traditional risk models to do not accurately predict short-term perioperative outcomes, other institutions who practice ERAS, have a large proportion of laparoscopic surgery and maintain prospective databases need to analyze the validity of the models tested in this study using their dataset.

## Conclusions

As time passes and surgical technique and postoperative recovery pathways reduce the physiological insult of surgery, we will find that the models based on these early data will loose validity. The existing perioperative risk prediction scores overestimate morbidity and mortality in patients undergoing colorectal surgery within an enhanced recovery program. New models are required based on prospectively collected data across multiple centers performing cases both laparoscopically and with enhanced recovery-based care this would fall in line more with the ACS-NSQIP database producing a more reliable and accurate tool for risk prediction in the UK setting.

## Electronic supplementary material

ESM 1 (DOCX 116 kb)
