# Supplementary material for: Perioperative risk prediction in the era of enhanced recovery: a comparison of POSSUM, ACPGBI, and E-PASS scoring systems in major surgical procedures of the colorectal surgeon
Source: Int J Colorectal Dis. 2018 Aug 4;33(11):1627–34. doi: 10.1007/s00384-018-3141-4 (PMC6208691; doi:10.1007/s00384-018-3141-4)
Supplement: Supplementary file 1 — (DOCX 116 kb) [file 384_2018_3141_MOESM1_ESM.docx]

**Appendix 1:** POSSUM, P-POSSUM, E-POSSUM physiological score (PS) and operative score (OS) parameters. Each respective algorithm is stated below.

|  | **Score** | | | |
| --- | --- | --- | --- | --- |
|  | **1** | **2** | **4** | **8** |
| **Physiological score (PS)** | | | | |
| Age | ≤60 | 60-69 | ≥70 |  |
| Cardiac signs | No failure | Diuretic, digoxin, angina or hypertension medication | Peripheral oedema or on warfarin | Raised central venous pressure or cardiomegaly |
| Respiratory signs | No dyspnoea | Mild COPD Dyspnoea on exertion | Moderate COPD. Dyspnea limits patients to single flight of stairs | Dyspneoa at rest. Fibrosis or consolidation |
| ECG | Normal |  | Rate controlled atrial fibrillation (60-90/min) | Any abnormal rhythm or ≥5 ectopics/minute or Q waves or ST/T wave changes |
| SBP (mmHg) | 110-130 | 131-170  100-109 | ≥171  90-99 | ≤89 |
| Pulse | 50-80 | 81-100 | 101-120 | ≥121 |
| Hb (g/100ml) | 13-16 | 11.5-12.9  16.1-17 | 10-11.4 | ≤9.9  ≥18.1 |
| WCC (x10^12^) | 4-10 | 10.1-20 | ≥20.1 |  |
| Urea (mmol/L) | ≤7.5 | 7.6-10 | 10.1-15 | ≥15.1 |
| Sodium (mmol/L) | ≥136 | 131-135 | 126-130 | ≤125 |
| Potassium (mmol/L) | 3.5-5 | 3.2-3.4  5.1-5.3 | 2.9-3.1  5.4-5.9 | ≤2.8  ≥6 |
| GCS | 15 | 12-14 | 9-11 | ≤9.9 |
| **Operative score (OS)** | | | | |
| Operative severity | Minor | Moderate | Major | Extended Major |
| Multiple procedures | 1 |  | 2 | >2 |
| Blood loss (ml) | ≤100 | 101-500 | 501-999 | ≥1000 |
| Peritoneal soiling | None | Minor (serous fluid) | Local pus | Free bowel content |
| Malignant disease status | None | Primary only | Nodal metastasis | Distal metastasis |
| Mode of surgery | Elective |  | Urgent | Emergency |

**POSSUM:** Log_e_ [R/1-R] = -7.04 + (0.13 x PS) + (0.16 x OS)

**POSSUM Morbidity:** Log_e_ [R/1-R] = -5.91 + (0.16 x PS)+(0.1 x OS)

**P-POSSUM:** Log_e_ [R/1-R] = -9.065 + (0.16 x PS) + (0.15 x OS)

**E-POSSUM:** Log_e_ [R/1-R] = -7.6942 + (0.1399 x PS)+(0.1126 x OS)

**E-POSSUM Morbidity:** Log_e_ [R/1-R] = -3526 + (0.0779 x PS) + (0.0949 x OS)

**Appendix 2:** CR-POSSUM physiological score (PS) and operative score (OS) parameters. The CR-POSSUM algorithm to derive mortality is stated below.

|  | **Score** | | | | |
| --- | --- | --- | --- | --- | --- |
|  | **1** | **2** | **3** | **4** | **8** |
| **Physiological score (PS)** | | | | | |
| Age | ≤60 |  | 61-70 | 71-80 | ≥81 |
| Cardiac failure | None/mild | Moderate | Severe |  |  |
| SBP (mmHg) | 100-170 | >170  90-99 | <90 |  |  |
| Pulse | 40-100 | 101-120 | >120 |  |  |
| Hb (g/100ml) | 13-16 | 10.-12.9  16.1-18 | <10  >18 |  |  |
| Urea (mmol/L) | ≤10 | 10.1-15 | >15 |  |  |
| **Operative score (OS)** | | | | | |
| Operative severity | Minor |  | Intermediate | Major | Extended Major |
| Peritoneal soiling | None/Serous | Local pus | Free pus or faeces |  |  |
| Operative urgency | Elective |  | Urgent |  | Emergency |
| Cancer stage | None. Dukes A/B | Dukes C | Dukes D |  |  |

**CR-POSSUM:** Log_e_ [R/1-R] = -9.167 + (0.33 x PS)+(0.30 x OS)

**Appendix 3: ACPGBI.** The individual scores of each of the four components (age, ASA, stage and urgency) are summated to give a total score, which is then used in the ACPGBI algorithm to calculate risk of mortality, stated below.

| **Age** | **ASA** | | **Stage** | **Urgency** |
| --- | --- | --- | --- | --- |
|  | **if cancer resected** | **if cancer not resected** |  |  |
| <65 = 0 | I = 0 | I = 1.7 | A/B = 0 | Elective = 0 |
| 65-74 = 0.7 | II = 0.8 | II =1.8 | C = 0.2 | Urgent = 0.8 |
| 75-84 = 1.1 | III = 1.6 | III = 2.1 | D = 0.6 | Emergency = 1.1 |
| 85-94 = 1.3 | IV = 2.5 | IV = 2.4 |  |  |
| >94 = 2.6 |  |  |  |  |

**ACPGBI score:** Log_e_ [R/1-R] = + (4.859 - total score)

**Appendix 4: E-PASS.** This score is dependent on pre-operative risk score (similar to physiological score for POSSUM) and surgical stress score (similar to operative score for POSSUM). The algorithm is stated below.

| **Pre-op risk score (PRS)** | | **Surgical stress score (SSS)** | |
| --- | --- | --- | --- |
| Age | Integer value in years | Blood loss/body weight | Integer value in ml/kg |
| Severe heart disease | No = 0, Yes = 1 | Operation time | Value measured in hours |
| Severe lung disease | No = 0, Yes = 1 | Surgery type | Open = 0, Laparoscopic = 1 |
| Diabetes | No = 0, Yes = 1 |  | |
| ECOG Performance status | Fully active = 0  Limited to light duties = 1  Active >50% day = 2  Confined to bed/chair >50% of waking day = 3  Completely dependent = 4 |  |  |
| ASA grade | Healthy = 1  Mild systemic disease = 2  Severe systemic disease = 3  Severe systemic disease that is constant threat to life = 4  Moribund = 5 |  |  |
| PRS = -0.0686 + (0.00345 x age) + (0.323 x heart disease) + (0.205 x lung disease) + (0.153 x diabetes) + (0.148 x performance status) + (0.0666 x ASA) | | SSS = -0.342+ (0.019 x blood loss/weight) + (0.0392 x op time) + (0.353 x surgery type) | |

**E-PASS score:** -0.328 + (0.936 x PRS) + (0.976 x SSS)

**Appendix 5: Supplementary Data (a) - Characteristics of included patients**

|  | | **Diagnosis** | | |
| --- | --- | --- | --- | --- |
|  |  | **IBD** | **Cancer** | **Other**  **Benign** |
| Number of operations | | 217 | 568 | 595 |
| Age | <60 | 194 | 124 | 292 |
|  | 60-69 | 15 | 180 | 144 |
|  | ≥70 | 5 | 271 | 155 |
| Gender | Male | 118 | 351 | 295 |
|  | Female | 96 | 224 | 296 |
| Surgical Approach | Laparoscopy | 159 | 476 | 421 |
|  | Open | 37 | 69 | 132 |
|  | Converted | 18 | 30 | 38 |
| ASA | 1/2 | 197 | 462 | 479 |
|  | 3/4 | 7 | 95 | 75 |
| UICC Stage | 0 |  | 2 |  |
|  | I |  | 160 |  |
|  | II |  | 197 |  |
|  | III |  | 163 |  |
|  | IV |  | 45 |  |
| Procedure | Segmental resection | 29 | 223 | 146 |
|  | Anterior resection | 31 | 179 | 76 |
|  | Abdominoperineal resection | 3 | 25 | 22 |
|  | Total or subtotal colectomy | 74 | 41 | 105 |
|  | Small bowel resection | 4 | 3 | 7 |
|  | Fashioning/closure of Stoma | 68 | 93 | 97 |
|  | Other^a^ | 5 | 11 | 138 |

a Other refers to any other colorectal procedures including reversal of Hartmanns’ operation, abdominal rectopexy, transanal resection, incisional hernia repair.

**Appendix 6: Supplementary Data (b): 30 day mortality, major complications, and complication as defined by POSSUM**

| **Outcome** | **Diagnosis** | | |
| --- | --- | --- | --- |
|  | **IBD** | **Cancer** | **Other Benign** |
| 30 day mortality | 0 | 7 | 4 |
| 30 day major morbidity (Clavien Dindo score 3-4) | 30 | 69 | 44 |
| **POSSUM complication:** definition | | | |
| **Wound haemorrhage:** local haematoma requiring evacuation | 0 | 0 | 0 |
| **Deep haemorrhage**: postoperative bleeding requiring re-exploration | 2 | 3 | 0 |
| **Chest infection:** purulent sputum with confirmed bacteriological cultures | 1 | 5 | 5 |
| **Wound infection:** wound cellulitis or the discharge of purulent exudate. | 8 | 26 | 17 |
| **Urinary infection:** >10^5^ bacteria per ml in previously clear urine | 4 | 12 | 7 |
| **Deep infection:** intra-abdominal collection confirmed clinically or radiologically | 7 | 5 | 4 |
| **Septicaemia:** confirmed by bacteriological positive blood cultures | 1 | 2 | 0 |
| **Wound dehiscence:** superficial or deep wound breakdown | 0 | 3 | 2 |
| **Deep venous thrombosis and pulmonary emboli:** confirmed radiologically or diagnosed at post mortem | 0 | 0 | 1 |
| **Cardiac failure:** signs and symptoms of left ventricular or congestive cardiac failure | 1 | 10 | 3 |
| **Renal impairment:** increase in serum urea of >5 mmol/l above preoperative levels | 0 | 2 | 1 |
| **Respiratory failure:** respiratory compromise requiring mechanical ventilation | 0 | 1 | 0 |
| **Anastomotic leak:** confirmed clinically or radiologically | 1 | 22 | 7 |

^a^ Data not available.
